# Genetic Modification of Tumor-Infiltrating Lymphocytes *via* Retroviral Transduction

**DOI:** 10.3389/fimmu.2020.584148

**Published:** 2021-01-07

**Authors:** Hadas Weinstein-Marom, Gideon Gross, Michal Levi, Hadar Brayer, Jacob Schachter, Orit Itzhaki, Michal J. Besser

**Affiliations:** ^1^ Ella Lemelbaum Institute for Immuno-Oncology, Sheba Medical Center, Ramat Gan, Israel; ^2^ Laboratory of Immunology, MIGAL-Galilee Research Institute, Kiryat Shmona, Israel; ^3^ Department of Biotechnology, Tel-Hai College, Upper Galilee, Israel; ^4^ Department of Clinical Microbiology and Immunology, Sackler School of Medicine, Tel Aviv University, Tel Aviv, Israel

**Keywords:** adoptive cell therapy, rapid expansion protocol, T cell subsets, T cell differentiation status, chimeric antigen receptors

## Abstract

Adoptive T cell therapy (ACT) holds great promise for cancer treatment. One approach, which has regained wide interest in recent years, employs antitumor T cells isolated from tumor lesions (“tumor-infiltrating lymphocytes” or TIL). It is now appreciated that a considerable proportion of anti-melanoma TIL recognize new HLA-binding peptides resulting from somatic mutations, which occurred during tumor progression. The clinical efficacy of TIL can potentially be improved *via* their genetic modification, designed to enhance their survival, homing capacity, resistance to suppression, tumor killing ability and additional properties of clinical relevance. Successful implementation of such gene-based strategies critically depends on efficient and reproducible protocols for gene delivery into clinical TIL preparations. Here we describe an optimized protocol for the retroviral transduction of TIL. As the experimental system we employed anti-melanoma TIL cultures prepared from four patients, recombinant retrovirus encoding an anti-CD19 chimeric antigen receptor (CAR) as a model gene of interest and CD19+ and CD19- human cell lines serving as target cells. Transduction on day 7 of the rapid expansion protocol (REP) resulted in 69 ± 8% CAR positive TIL. Transduced, but not untransduced TIL, from the four patients responded robustly to CD19+, but not CD19- cell lines, as judged by substantial secretion of IFN-γ following co-culture. In light of the rekindled interest in antitumor TIL, this protocol can be incorporated into a broad range of gene-based approaches for improving the in-vivo survival and functionality of TIL in the clinical setting.

## Introduction

ACT with autologous TIL produced from resected melanoma biopsies combined with non-myeloablative lymphodepletion and IL-2 exceeds an overall clinical response rate of 41% [95% confidence interval (CI) 35% to 48%] in patients with highly advanced metastatic melanoma according to meta-analysis (1–5). The TIL protocol consists of three basic steps: (i) patient preconditioning by lymphodepleting regimen; (ii) Isolation of TIL from tumor biopsies and ex-vivo expansion of generated TIL cultures with the anti-CD3 antibody OKT3, irradiated feeder cells and IL-2; (iii) TIL administration i.v. followed by IL-2 infusion.

We have been applying for over a decade an open label phase II clinical study in stage IV melanoma patients (2, 6–11). Our initial objective was to assess the efficacy and safety of ACT with autologous TIL, which was offered as a second line of treatment following IL-2–based therapy until 2011 and, since, as a salvage treatment following standard of care therapy including other immunotherapy modalities (e.g., anti-CTLA-4 and anti-PD-1 antibodies). Following surgical resection and processing of a metastatic lesion, TIL cultures are typically generated in IL-2–containing medium within 2 weeks to 1 month. Established TIL cultures are then further expanded in a rapid expansion protocol (REP) to treatment levels. REP is initiated with an anti-CD3 agonist, irradiated feeder cells and IL-2, which drives the expansion of TIL to approximately 1000-fold within 14 days. Clinical results of 57 patients treated at our medical center have previously been published (10). Potential predictors for response were shorter time to TIL culture generation, elevated fold large scale expansion, higher total number of cells, higher total number of CD8 T cells and higher frequency of CD8 T cells in the cell product (10).

In spite of the tremendous progress achieved in TIL therapy, tumor specimens may fail to give rise to viable TIL cultures and a high proportion of melanoma patients treated with either TIL-ACT strategy are non-responders (5, 12, 13). Although many responders exhibit long-lasting tumor regression and complete responders only rarely relapse, new strategies are needed for maximizing response rate and duration and, most importantly, for providing a platform for the treatment of other solid tumors. Undoubtedly, a promising route for improving the clinical efficacy of TIL therapy is the enhancement of the functional properties of TIL cultures *via* genetic modification. To achieve this goal we have recently created several classes of “genetic adjuvants,” including membrane-attached cytokines, truncated, constitutively active toll-like receptors (caTLRs) and spontaneously homo-oligomerizing, constitutively active tumor-necrosis factor receptors (caTNFRs). Using electroporation of anti-melanoma TIL with in-vitro-transcribed mRNAs encoding membrane IL-2, IL-12, IL-15, caTLR4, or caCD40, alone or in different combinations, we could demonstrate their additive, and often synergistic effects on key parameters concerning TIL function and survival (14–18).

The first clinical assessment of this approach was reported by S. Rosenberg et al. (19) who had successfully introduced the neomycin-resistance gene into anti-melanoma TIL derived from five patients *via* retroviral transduction. Gene-expression in modified TIL could be detected in the circulation of these patients three weeks to two months post-administration. In following studies, the Rosenberg group demonstrated the retroviral transduction of anti-melanoma TIL with the IL-2 gene (20) or with an inducible IL-12 gene placed under the control of a nuclear factor of activated T cells- (NFAT)-responsive promoter (21). While all these studies employed selected anti-melanoma TIL, Forget et al. recently described a protocol for the retroviral transduction of TIL, evaluating the gene encoding the chemokine receptor CXCR2 (22). Here we describe in detail our optimized protocol for retroviral transduction of anti-melanoma TIL cultures and demonstrate its efficacy in endowing transduced TIL with a new antigenic specificity *via* an anti-CD19 CAR.

## Materials and Methods

### Patient Samples, Retroviral Vector, and Cell Lines

TIL samples were obtained from melanoma patients that were enrolled to an open-label and phase II TIL ACT trial for patients with metastatic melanoma at the Sheba Medical Center (NCT00287131). A gamma-retrovirus encoding an anti-CD19 CAR based on an FMC63 derived scFv, a CD28 costimulatory domain and CD3-ζ signaling domain, as well as the following CD19-expressing immortalized cell lines were used: NALM-6 (acute lymphoid leukemia); Toledo (B cell diffuse large cell lymphoma); CD19-K562 cells (CD19-transduced K562 chronic myeloid leukemia cells). NGFR-K562 cells (NGFR- (nerve growth factor receptor)-transduced K562), served as CD19-negative control cells. All cell lines and the retroviral vector were kindly provided by Dr. S. Feldman, NCI. Vector construction and retroviral vector master cell bank production were done at the NCI.

Peripheral blood mononuclear cells (PBMC) of healthy donors (HD) or a lymphoma patient (CAR-T Pt.) were prepared from apheresis products collected at the Sheba bone marrow transplantation unit by single step gradient with Ficoll-Hypaque in lymphocyte separation medium.

### Establishment of TIL Cultures

TIL establishment was previously described by us in detail (8–10). In short, fragmentation, enzymatic digestion and cell remnants technique were used to isolate TIL from surgically resected metastatic lesions. During the first week, non-adhered TIL were transferred to a new 24-well plate and cultured separately from the adhered melanoma cells. Cells were cultured in complete medium (CM) containing 10% human AB serum (Valley Biomedical, Winchester, VA or Gemini Bio, West Sacramento, CA), 2 mM l-glutamine (Biological Industries, Israel), Pen/Strep (Biological Industries, Israel) in RPMI 1640 medium (Gibco, Thermo Fisher Scientific, Waltham, MA). 3,000 IU/ml IL-2 (Chiron Novartis, NJ, USA) was added to the CM medium during the TIL establishment phase. Cells were split or medium was added every 2 to 3 days to maintain a cell concentration of 0.5–2 × 10e6 cells/ml. TIL cultures were established within 2 to 4 weeks.

### Establishment of Melanoma Cultures

The generation of melanoma cell lines was previously described (8–10). Enzymatic digestion and cell remnants technique were used to isolate melanoma cells from surgically resected metastatic lesions. Melanoma cells were cultures in Target medium contained 10% Fetal Bovine Serum, 25 mmol/L HEPES, 100 U/ml penicillin, 100 μg/ml streptomycin and 1 mM sodium pyruvate (Biological Industries, Israel).

### Standard CAR Protocol (SCP)

This process describes a smaller scale version of our standard expansion protocol of CAR T cells for clinical use (23). On the day of SCP initiation 16×10^6^ PBMC or two days prior SCP initiation 8×10^6^ TIL were thawed in CM medium with 3,000 IU/ml IL-2. On day 0 of SCP cells were re-suspended at a concentration of 1.0×10^6^ cells/ml in Stim medium containing 5% human AB serum (Valley, VA, US), 2 mM l-glutamine (Biological Industries, Israel), 100 U/ml penicillin and 100 mg/ml streptomycin (Biological Industries) in AIM-V medium (Invitrogen, CA, USA, CM). 300 IU/ml IL-2 (Chiron Novartis, NJ, USA) and 50 ng/ml anti-CD3 monoclonal antibody OKT3 (Miltenyi Biotec, Bergisch Gladbach, Germany) were added. After 2 days, 2 × 10^6^ cells were transduced with the CD19 CAR retroviral vector and the rest of the cells discarded. For this purpose, non-tissue culture treated 6-well plates were coated with 10 µg/ml RetroNectin (Takara Bio Inc, Otsu, Japan) in DPBS-Dulbecco Phosphate Buffered Saline (Biological Industries, Israel) for 2 h at room temperature or overnight at 4°C, followed by 30 min blocking with 2.5% human albumin (Bio Products Laboratory Limited, UK) in PBS and washed. Retroviral supernatant was thawed and diluted 1:1 with Stim medium. Four ml of the diluted vector were added per well of the retronectin-coated plates and these were centrifuged at 2,000*g* for 2 h at 32°C. Supernatant was aspirated and 2 × 10^6^ TIL or PBMC in Stim medium with 300 IU/ml IL-2 were added to each well, centrifuged for 10 min at 1,000*g* and incubated at 37°C overnight. On day 3 the transduced cells were transferred to 6-well culture plates and maintained at a concentration of 0.5–2.0 × 10^6^ cells/ml in Stim medium with 300 IU/ml IL-2 and further expanded until day 10, the standard day of CAR T cell infusion.

### Rapid Expansion Protocol (REP) With Viral Transduction

This process describes the standard rapid expansion protocol of TIL cells for clinical use, just in a smaller scale (9, 10). TIL were thawed in CM with 3,000 IU/ml IL-2 and allowed to rest for a period of 2 days at a concentration of 1.0×10^6^/ml in a 24-well plate. A mini-scale REP was initiated by stimulating TIL with 30 ng/ml OKT3, 3,000 IU/ml IL-2 and irradiated PBMC from non-related donors as feeder cells (5000 rad, TIL to feeder cells ratio = 1:100) in 50% CM and 50% AIM-V medium in T25 flasks. On the day of viral transduction (REP day 7), TIL were harvested, counted and adjusted to a concentration of 0.5×10^6^/ml in CM with 3,000 IU/ml IL-2. Four ml of the cell suspension was distributed per well of a 6-well plate layered with viral vector as follows: Plate coating and viral transduction were performed as described above for the SCP. The viral supernatant was then aspirated and 2 × 10^6^ TIL in Stim medium with 3,000 IU/ml IL-2 were added to each well, centrifuged for 10 min at 1,000*g* and incubated at 37°C overnight. The next day the cells were transferred to T75 culture flasks and maintained at a concentration of 0.5–2.0 × 10^6^ cells/ml in Stim medium with 3,000 IU/ml IL-2 until day 14 (potential day of TIL infusion).

### Flow Cytometry

Transduction efficacy was determined 4 days after transduction (day 11 of REP and day 6 of SCP) and 7 days after transduction (day 14 of REP and day 9 of SCP) using flow cytometry by labeling CAR-expressing TIL with biotin-labeled, goat anti-mouse IgG, F(ab′)2-specific antibody (Jackson Immunoresearch, West Grove, PA) and streptavidin (APC conjugated; BD Bioscience, San Jose, CA). The following anti-human antibodies were used in addition: anti-CD3 (Pacific-Blue conjugated, BioLegend, San Diego, CA), anti-CD4 (FITC-conjugated; BioLegend) anti-CD8 (PE-cy7-conjugated; BioLegend or APC conjugated; BD Biosciences), anti CD28 (PerCP vio770 conjugated, Miltenyi Biotec), anti-LAG-3 (Vioblue conjugated; Miltenyi Biotec), anti-PD1 (FITC conjugated, BioLegend), and TIM-3 (PE-cy7 conjugated; BioLegend). CD3+/F(ab′)2+ cells were defined as “transduced” cells. For further analysis of memory cell phenotype, cells were stained with antibodies to CD45RA (APC-Vio770-conjugated; BioLegend), CCR7 (PerCP-Vio770 conjugated; BioLegend). TIL were washed and resuspended in cell staining buffer (BioLegend, San Diego, CA). Cells were incubated for 30 min with the antibodies on ice, washed in buffer, and measured using FACS cytometer MACSQuant (Miltenyi Biotec). Samples were analyzed using FlowJo software (FlowJo LLC, Ashland, OR). Cells were gated according to FCS/SSC and singlets. The gating strategy is shown in **Supplementary Figure 1**.

### Interferon-γ Release Assay

TIL were co-cultured overnight with target cells in 96-well plates at an E/T ratio of 1:1 (5 × 10^5^ each in a total of 200 μl Target medium). Cells were centrifuged, supernatant was collected, and secreted IFN-γ levels were determined by a sandwich ELISA according to the manufacturer’s instructions (BioLegend). Measurements were performed in triplicates.

### Cell-Mediated Cytotoxicity Assay

TIL were co-cultured with autologous melanoma cells overnight at 37°C, at an E/T ratio of 1:1 (1 × 10^5^ each in a total of 200 μl Target medium. Levels of lactate dehydrogenase (LDH) in the medium were determined by CytoTox 96 Non-Radioactive Cytotoxicity Assay (Promega, Madison, WI, Cat. no. G1780) according to the manufacturer’s instructions, using a microplate reader (Antos 2000) at 490 nm. Experiments were performed in triplicates wells. Percent of specific lysis of target cells was calculated using the equation: (Experimental-EffectorSpontaneous − TargetSpontaneous)/(TargetMaximum − TargetSpontaneous) × 100.

### Statistical Analysis

Significance of variation between groups was evaluated using a non-parametric two-tailed Student’s t test. Test for differences between proportions was performed using two sided Fisher’s exact test with p ≤ 0.05 considered significant.

## Results and Discussion

### TIL Transduction During SCP

As an indicator gene for demonstrating TIL transduction efficacy we have chosen the same anti-CD19 CAR we use for treating patients with B cell malignancies (24) rather than, for example, an anti-melanoma CAR. We reasoned that monitoring the newly acquired activity of the anti-melanoma TIL against non-melanoma cells would allow us to determine the net contribution of the CAR gene on TIL function with no background activation or potential bottlenecks along the signaling pathway.

TIL cultures were established from tumor biopsies of melanoma patients within two to three weeks as described before (8–10).

**Figure 1:**
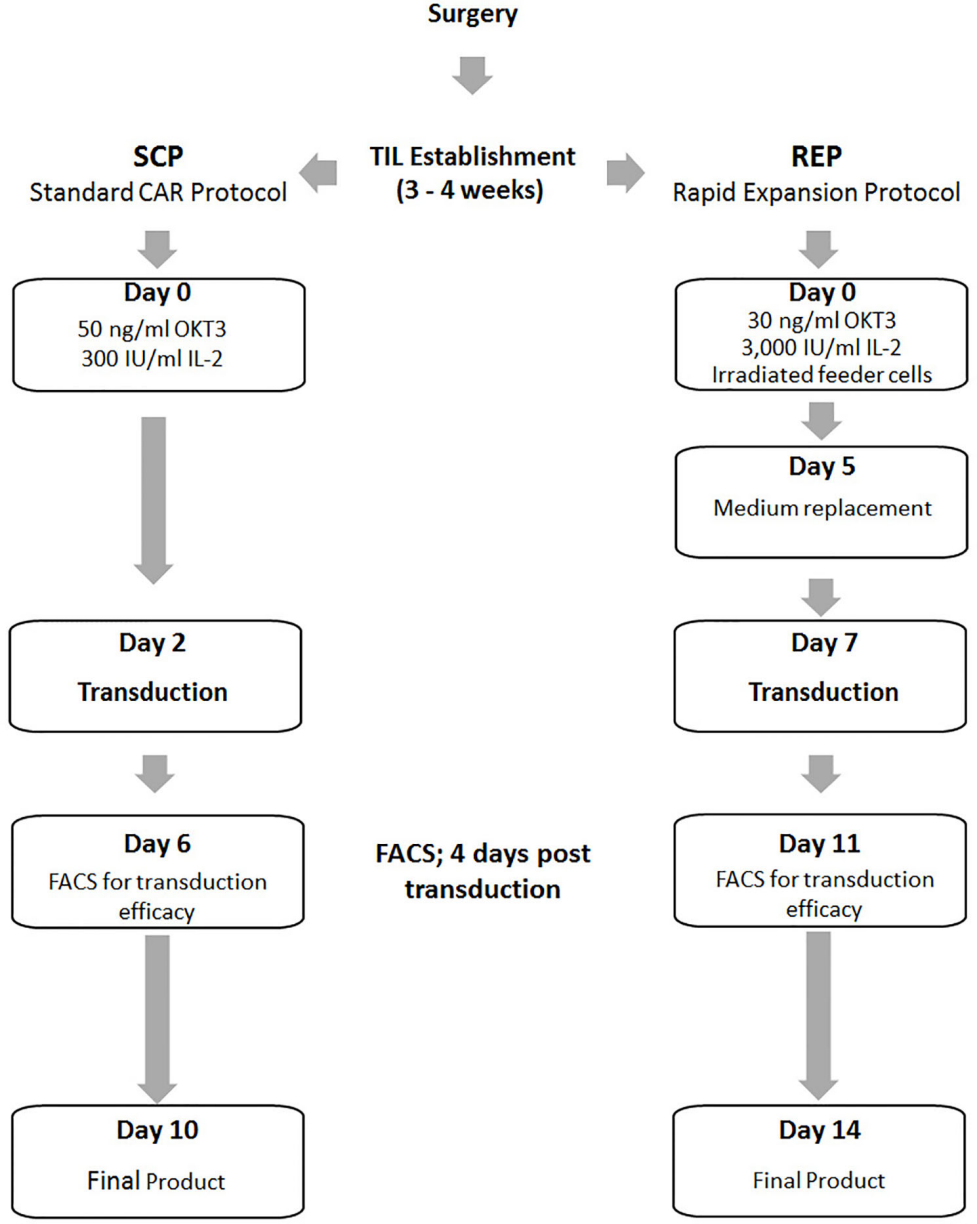
Schematic illustration of the Standard CAR Protocol (SCP) compared to the Rapid Expansion Protocol (REP).

With the aim to develop an optimized TIL transduction method, we first utilized a standard CAR-T cell production protocol (SCP) (**Figure 1**, left panel). In the clinical setting, CAR T production is initiated with PBMC. Here we applied the exact same protocol on TIL cultures derived from three melanoma patients (TIL052, TIL189, and TIL213) and compared fold expansion, transduction efficacy, cell phenotype to results obtained from three healthy donors (HD) PBMC. In short, TIL or HD PBMC were stimulated with OKT3 and IL-2 (23). On day 2, 2 × 10^6^ TIL or PBMC were transduced with an anti-CD19 CAR construct on retronectin-coated plates and further expanded in IL-2-containing medium until day 10, the standard day of infusion.

**Figure 2:**
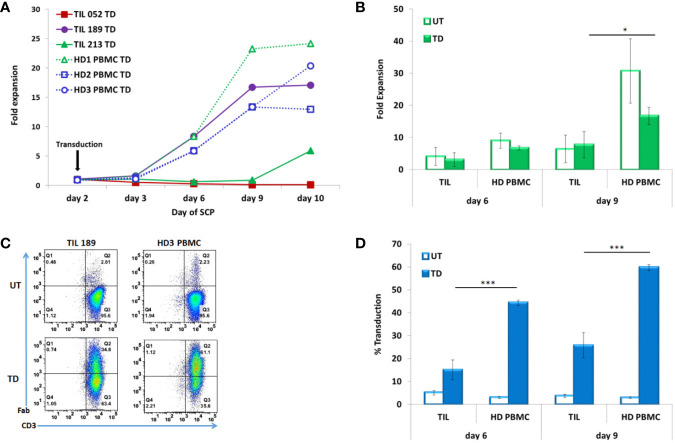
TIL compared to PBMC transduced according to the SCP. **(A)** Fold expansion after transduction (day 2) of melanoma patients derived TIL (TIL052, TIL189, and TIL213) compared to healthy donor (HD) PBMC **(B)** Average fold expansion of transduced (TD) and untransduced (UT) TIL and HD PBMC on days 6 and 9. **(C)** Transduction efficacy determined by CD3/F(ab′)2 double positive cells, representative FACS histogram plot **(D)** Average frequency of transduced TIL and PBMC on days 6 and 9. *p < .05, ***p < .001.

Cell counts were performed on days 3, 6, 9, and 10. **Figure 2A** presents the fold expansion over time compared to day 2, the day of CD19 CAR transduction. As shown in **Figure 2B**, HD PBMC expanded better than TIL: fold expansion on day 6 was 6.71 ± 0.68 for HD PBMC and 3.11 ± 2.15 for TIL (p = .0510) and on day 9, 16.67 ± 2.7 for HD PBMC and 7.71 ± 4.06 for TIL (p = .0334)

**Figure 3:**
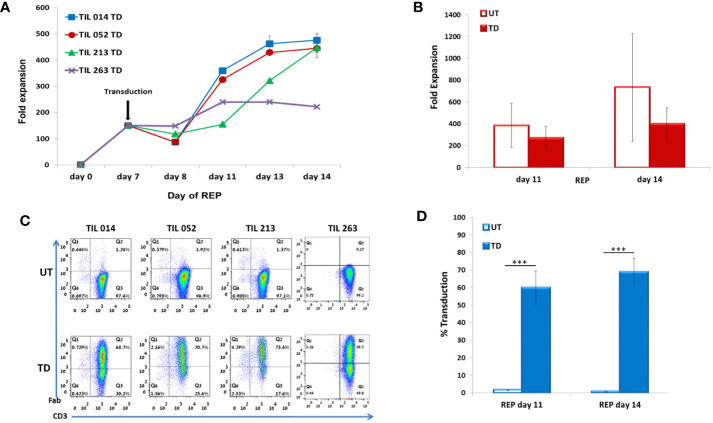
Characterization of REP/d7-transduction protocol. TIL014, TIL052, TIL213, and TIL 263 cultures were transduced with the anti-CD19 CAR on day 7 of REP. **(A)** Fold expansion during REP. **(B)** Average fold expansion of transduced (TD) and untransduced (UD) TIL on days 11 and 14 of REP. **(C)** Transduction efficacy as determined by flow cytometry analysis for the CD3+F(ab′)2+ expression; representative FACS histogram plot. **(D)** Average frequency of transduced cells on days 11 and 14 of REP. ***p < .001.

Transduction efficacy was determinate by F(ab′)_2_ expression on CD3 T cells on day 6 (4 days after transduction) and day 9 (7 days after transduction). A representative FACS dot plot is shown in **Figure 2C**. A minor fraction of untransduced TIL and PBMCs were also stained by the same biotin-labeled polyclonal whole goat IgG anti-mouse F(ab′)_2_ antibody, as can also be seen in **Figure 3C**. This staining could be accounted for a small subset of human CD4 T cells naturally expressing FcγRI (25). Transduction efficacy was significantly higher in HD PBMC than in TIL (day 6: 44.4 ± 1.17% for HD PBMC and 15.08 ± 4.24% for TIL, p = .0003; day 9: 59.73 ± 1.26% for HD PBMC and 25.77 ± 5.46% for TIL, p = .0005) (**Figure 2D**). Due to the superior expansion and elevated transduction efficacy in HD PBMC compared to TIL, the total number of transduced cells by day 10 was 10.8 fold higher in HD PBMC than in TIL (2.42 × 10^7^ ± 8.67 × 10^6^ vs. 2.24 × 10^6^ ± 2.91 × 10^6^, p = .0141). This phenomenon could reflect inefficient TIL stimulation at the TIL establishment stage in the absence of feeder cells and might correlate with low expression of the co-stimulatory molecule CD28 [(26), see **Table 1**].

**Table 1:** Phenotypic profile of untransduced (UT) vs. transduced (TD) TIL and HD PBMC on day 9 of SCP. Data are presented as average % expression of the indicated markers.

	TIL			HD PBMC		
	UT (n=3)	TD (n=3)	p value	UT (n=3)	TD (n=3)	p value
**CD3+**	99 ± 0.9	99 ± 0.6	p = 1.00	99 ± 0.2	99 ± 0.4	p = 1.00
**CD4+**	40 ± 34	45 ± 34	p = .866	42 ± 11	39 ± 13	p = .776
**CD8+**	60 ± 34	55 ± 34	p = .866	58 ± 11	61 ± 13	p = .776
**T_N_**	6.0 ± 5.3	11 ± 3.6	p = .248	14 ± 4.3	15 ± 2.7	p = .750
**T_CM_**	19 ± 6.6	14 ± 8.3	p = .460	21 ± 4.7	21 ± 4.1	p = 1.00
**T_EM_**	70 ± 13	63 ± 11	p = .516	54 ± 11	50 ± 2.3	p = .571
**T_EMRA_**	5.0 ± 0.7	9.0 ± 2.8	p = .074	12 ± 2.7	14 ± 3.2	p = .455
**CD28+CD3+**	13 ± 12	5.0 ± 4.0	p = .335	21 ± 19	8.0 ± 6.0	p = .322
**LAG3+CD3+**	3.0 ± 4.0	3.0 ± 2.0	p = 1.00	1.0 ± 0.2	5.0 ± 2.0	p = .026
**PD1+CD3+**	31 ± 7.0	31 ± 8.0	p = .925	5.0 ± 2.0	6.0± 2.0	p = .725
**TIM-3+CD3+**	40 ± 17	25 ± 13	p = .306	57 ± 10	50 ± 10	p = .395

TN (naïve), CD3+CD45RA+CCR7+; TCM (central memory), CD3+CD45RA−CCR7+; TEM (effector memory), CD3+CD45RA−CCR7−; TEMRA (effector), CD3+CD45RA+CCR7−.

T cell subset distribution and differentiation status have been related to clinical response both in CAR-T and TIL therapies (27–29). Additional phenotype analysis performed on transduced and untransduced TIL on day 9 revealed that transduction had no impact on the T cell CD4/CD8 distribution. T cell differentiation status (examining naïve, central memory, effector memory and terminally differentiated effector T cells (T_N_, T_CM_, T_EM_, and T_EMRA_, respectively) and the expression of the co-stimulatory molecule CD28 or co-inhibitory molecules LAG-3, TIM-3, and PD-1 (**Table 1** and **Supplementary Figure 2**) were also preserved. With the exception of increased LAG-3 expression on transduced HD PBMC (UT, 1.0 ± 0.2%; TD, 5.0 ± 2.0%; p = .0261), transduction had also no impact on the phenotype of HD PBMC (**Table 1**).

### TIL Transduction During REP

Since SCP resulted in a low transduction efficacy and expansion, we tested if transduction during TIL REP could yield superior results (**Figure 1**, right).

TIL expansion during the establishment phase is driven by the addition of IL-2 without direct TCR activation. During REP, we routinely provide the cells with optimal signaling *via* three complementary pathways: Cell proliferation is induced by IL-2, T cell activation is triggered by an anti-CD3 antibody (OKT3) and co-stimulation is delivered by feeder cells. In this study we did not assess the separate contribution of each of these stimuli to TIL expansion or transduction efficacy. We used soluble OKT3 in both SCP and TIL REP as we routinely do in our clinical trials with CAR-T cells and TIL. In both protocols, delivering highly proliferative cells is critical for clinical outcome. Indeed, comparison of CD3/CD28 beads to soluble OKT-3 and high concentrations of IL-2 showed that the latter produced more T_EM_ cells with shorter telomeres (30). Yet, employing our protocol we achieved high transduction efficacy during REP, which reflects the ability of soluble OKT3/IL-2 in the presence of feeder cells to induce potent activation of TIL possessing enhanced proliferative capacity.

In contrast to SCP, which results in an average expansion of around 20-fold on day 10 (23), transduction during REP results in about 1,000-fold expansion after 14 days (9). In the clinical setting, day 14 is the day of cell infusion. Based on our previous experience with TIL ACT, at days 6 to 8 of REP, cells possess a high proliferation capacity while the presence of feeder cells, which may decrease transduction yield, is highly reduced. We first demonstrated transduction efficacy of ~70% in REP for day 6 and day 8 cultures (TIL 052: day 6, 71.8 ± 1.4%; day 8, 69.4 ± 0.8%; p = .18). As a compromise between the higher transduction efficacy observed for day 6 and day 8 REP, and the likelihood for remaining feeder cells in the culture we have chosen REP day 7 as the optimal transduction day, as no more irradiated donor feeder cells were present in the culture at that time point. Transduction was performed with TIL samples derived from four melanoma patients (TIL014, TIL052, TIL213, and TIL263).

TIL were counted on days 7, 8, 11, 13 and 14 (7 days post-transduction). Fold expansion over time is presented in **Figure 3A**. Although untransduced TIL expanded significantly better than CD19 CAR-transduced TIL (day 11: UT 383 ± 201, TD 270 ± 104, p = .3567; day 14: UT 734 ± 494, TD 397 ± 149, p = .2408), the average fold expansion of transduced TIL on day 14 reached 397 ± 149 (**Figure 3B**).

Transduction efficacy was determined 4 days and 7 days (day 11 and day 14 of REP) after viral transduction (**Figure 3C**). As shown in **Figure 3D**, the average frequency of transduced TIL was 60 ± 9.6% on day 11 of REP and 69 ± 8% on day 14 of REP. Of note, the transduction efficacy achieved 4 days following transduction during REP was significantly higher than during SCP (REP 69 ± 8%, SCP 15 ± 4.2%; p <.0001). Transduction did not affect the CD8/CD4 subset ratio but had an effect on the differentiation status of TIL (**Table 2**).

**Table 2:** Phenotypic profile UT vs. TD TIL undergoing transduction during REP.

	TIL		
	UT (n=4)	TD (n=4)	p value
**CD3+**	100 ± 0.05	100 ± 0.1	p = 1.000
**CD4+**	15 ± 4.7	16 ± 5.4	p = .7893
**CD8+**	85 ± 4.7	84 ± 5.4	p = .7893
**T_N_**	5.6 ± 3.9	5.5± 2.7	P = .9677
**T_CM_**	12 ± 1.3	21 ± 4.1	P = .0058
**T_EM_**	76 ± 4.4	64 ± 6.5	p = .0223
**T_EMRA_**	5.9 ± 2.9	9.8 ± 3.2	p = .1209

Data is presented as average % expression of the indicated markers. TN (naïve), CD3+CD45RA+CCR7+; TCM (central memory), CD3+CD45RA−CCR7+; TEM (effector memory), CD3+CD45RA−CCR7−; TEMRA (effector), CD3+CD45RA+CCR7−.

**Figure 4:**
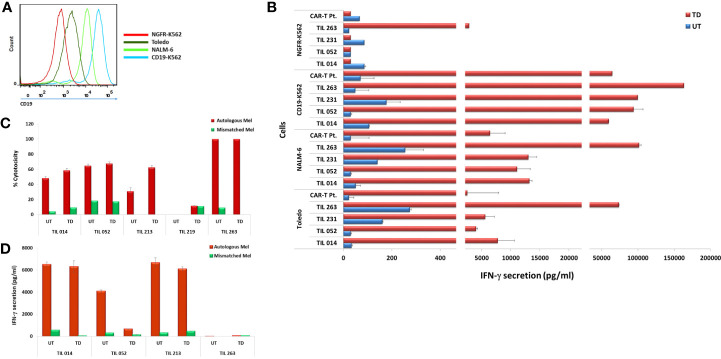
Transduced CAR-TIL are reactive against CD19-expressing cell lines and autologous melanoma cultures. **(A)** Flow cytometry analysis of target cell lines for CD19 expression. **(B)** Transduced (TD) and untransduced (UT) TIL were co-cultured for 24 h with target cells expressing CD19 (K562-CD19, Toledo, NALM-6) or not (K562-NGFR) followed by IFN-γ ELISA. CAR-T Pt. cells served as a positive control. **(C)** Transduced (TD) and untransduced (UT) TIL were co-cultured with autologous melanoma cells overnight at 37°C at an E/T ratio of 1:1. Growth medium was then removed and subjected to an LDH non-radioactive cytotoxicity assay. Experiments were performed in triplicates wells. **(D)** Transduced (TD) and untransduced (UT) TIL were co-cultured for 24 h with autologous melanoma target cells followed by IFN-γ ELISA. IFN-γ secretion by target cell lines alone, T cells alone, and T cells co-cultured with HLA-mismatched melanoma cell lines were below the level of detection and are not shown.

The four CD19 CAR-transduced TIL cultures were further analyzed for antitumor reactivity by assessing IFN-γ secretion in response to CD19 stimulation. To this end, TIL were co-cultured with the CD19-expressing cell lines TOLEDO, NALM-6, and CD19-K562 and the CD19-negative cell line NGFR-K562 (**Figure 4A**). Untransduced TIL served as negative control and T cells transduced with the same CAR construct of a lymphoma patient who achieved complete clinical remission following CAR-T cell therapy served as positive control (CAR-T Pt.; transduction efficacy: 60.1%). Transduced TIL demonstrated robust anti-tumor reactivity to the three CD19-positive targets as monitored by IFN-γ secretion, (CD19-K562 = 59,708–163,344 pg/ml; Nalm-6 = 11,081–101,483 pg/ml; Toledo, = 5,591 - 73,974 pg/ml), which was comparable, and often superior to transduced cells of the clinical responder. Practically no response was seen against the CD19 negative-cells NGFR-K562 except for TIL263 that showed secretion of 2,839 ± 373 pg/ml compared with the significantly higher secretion against CD19-K562 positive target (163,344 pg/ml) (**Figure 4B**).

A critical component of TIL anti-melanoma response is the ability to kill tumor cells directly. We compared the ability of transduced TIL to respond to their autologous melanoma or unrelated, HLA class-I-mismatched melanoma with that of untransduced cells as judged by target cell killing. As shown in **Figure 4C**, the four TIL maintained their specific killing of autologous melanoma. Transduced TIL014, TIL052, and TIL213 showed an increased killing activity compared to the untransduced cells, and TIL263 maintained its original 100% killing activity achieved in this assay.

We also assessed IFN-γ secretion of four TIL in response to their autologous melanoma. As shown in **Figure 4D**, TIL014 and TIL213 retained their ability to secrete high amount of IFN-γ. TIL052, however, exhibited considerable reduced secretion, although IFN-γ level of 660 pg/ml still exceeds the acceptance criterion of 200 pg/ml that is implemented in selected TIL procedures (2, 7). The reason for this reduction is not clear. Considering the apparent clonal diversity of the initial TIL052 culture and the cell-specific events of viral genome integration, a direct negative effect of transduction on TIL function as seen here is unlikely. TIL263 secreted only little IFN-γ in response to the autologous melanoma even prior to transduction.

All TIL chosen for this study are short-term-cultured or “young” TIL, not selected according to their ability to secrete IFN-γ in response to their autologous tumor (8, 31). In our own experience, only approximately half of the patients have TIL capable of IFN-γ secretion exceeding the acceptance criterion of 200 pg/ml required for selected TIL. Yet, a considerable fraction of non-secreting TIL are still capable of efficient target cell killing, as clearly demonstrated here with TIL263.

**Figure 5:**
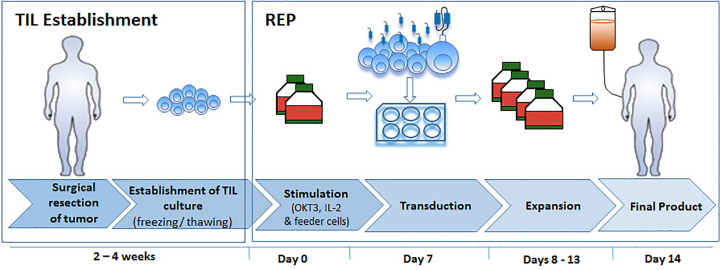
Flowchart of optimized TIL expansion and transduction.

To summarize our protocol for retroviral TIL transduction, a scheme of the workflow is presented in **Figure 5**: Following the establishment of TIL cultures and cryopreservation, TIL are thawed and allowed to rest for 2 days before REP initiation. On day 0 of REP, TIL are re-suspended in CM/AIM-V medium with soluble anti-CD3 antibody, IL-2, and irradiated feeder cells. Transduction is performed on day 7, followed by the expansion of the transduced cells until day 14, the planned day of infusion. The transduction protocol did not exert a discernible effect on the CD8/CD4 T cell subset ratio and the differentiation status of TIL cultures. This protocol results in a transduction efficacy of ~ 69% and an average expansion of > 400-fold. Although transduction reduces TIL proliferation capacity by approximately two-fold compared with untransduced cells (**Figure 3B**), the protocol is highly efficient and achieves clinically relevant numbers of genetically modified cells.

Genetically engineered TIL with improved functionality and/or tumor recognition capacity hold great promise in adoptive cell therapy and several laboratories have already provided detailed protocols for retroviral TIL transduction.

This field was pioneered by S. Rosenberg and colleagues, who in 1990 introduced a neomycin-resistance gene to anti-melanoma TIL derived from five patients, to allow the identification of transductants and their offspring in long-term studies of patients (19). In that work, transduction was performed during TIL establishment, i.e., at the pre-REP phase, and entailed lengthy ex-vivo propagation of up to 65 days, which may often result in T cell exhaustion. Transduction efficacy in TIL cultures, which was determined by semi quantitative (sq) PCR, did not exceed 11% and was comparable to the results obtained by a quantitative Southern blot analysis that revealed 4% to 18% efficacy. This work was the first demonstration of the feasibility and safety of human gene therapy through retroviral transduced TIL. In a later study the Rosenberg group investigated ACT of 13 patients who were treated with anti-melanoma TIL transduced with the IL-2 gene employing three different protocols (20). In cohort I (three patients), transduction was performed twice at the pre-REP stage, resulting in ~1000 fold expansion on day 14 and an estimated efficacy of ~21% (as determined by sqPCR). In cohort II and III (five patients each), transduction was performed during first rapid expansion, which was followed by a second expansion phase. TIL of cohort II were transduced twice, on days 7 and 8, and in cohort III TIL were transduced on days 3 and 4. The results for cohort II and III demonstrated ~25% to 35% transduction yield as judged by intracellular staining for IL-2 performed day 14. In comparison, our REP day 8 transduction achieved ~68% efficacy, as monitored by flow cytometry analysis.

Importantly, the lengthy ex-vivo expansion period may have contributed to shorter telomeres, potentially limiting in-vivo survival. In a third report (21), the Rosenberg group reported the transduction of TIL from 33 patients with an inducible IL-12 gene, employing either a dual transduction protocol on REP day 4 and 5 for 21 patients and a single transduction on either day 4 or day 5 for 12 patients. Gene transfer efficiency ranged from 2% to 52%, averaging 14%, with generally diverse, but low expansion rate at day 14.

We observed an average of two-fold reduction in the proliferation capacity of transduced vs. untransduced TIL, which might reflect the increase in T_CM_ and decrease in T_EM_ phenotype in transduced cells (**Table 2**). T_CM_ and T_EM_ cells comprised 12 ± 1.3% and 76 ± 4.4, respectively, in the untransduced TIL compared to 21 ± 4.1% and 64 ± 6.5% in the transduced cells (p < .0058 and p < .0223, respectively). Our results are in agreement with those of Zhang et al. (21). Nevertheless, T_CM_ represents a population with self-renewal capability, which can be advantageous in ACT as cells derived from the T_CM_ population were shown to persist and expand in-vivo better than those derived from T_EM_ cells (29, 32).

More recently, Forget et al. (22) transduced four anti-melanoma TIL with the CXCR2 gene with transduction efficiency averaging 45%. In their study, transduction was performed on day −1 before REP in the presence of plate-bound, rather than soluble OKT3 at different concentrations and 2 periods of activation of 24/48 h. TIL from one patient contained a considerable proportion of double-negative CD3+ cells that did not expand. In our transduction protocol (see **Figure 5**) we stimulate the cells only during the REP stage (with 30 ng/ml soluble OKT3). Our protocol supports the growth of TIL populations, which comprise exclusively single-positive CD3+ (**Table 2**) and maintains proliferative capacity. Tumor infiltration of CD3+CD8+ T cells is associated with a higher rate of progression-free survival and improved prognosis (5).

Compared to transduction during the TIL establishment phase [as practiced in (22)], transduction during REP requires a greater number of cells, but leads to higher transduction efficacy. It is conceivable that improvement in clinical efficacy achieved by the delivered gene could eventually enable lowering the number of administrated TIL, which ranges between 0.5 × 10^10^ and 10 × 10^10^ cells per infusion.

With the growing appreciation of the importance of neo-antigens in the induction of the antitumor response, the field of TIL ACT has been gaining even greater interest in the past several years [e.g., (33–35)]. Here we used TIL transduction with a CD19 CAR as an experimental model. However, this method is not limited to the introduction of an additional antigen receptor and can be used for the delivery of genes designed to prolong TIL survival, confer resistance to suppression, enhance different functional properties, and increase homing to the tumor site, all of which can improve the clinical efficacy of TIL therapy.

## Data Availability Statement

The raw data supporting the conclusions of this article will be made available by the authors, without undue reservation.

## Ethics Statement

The studies involving human participants were reviewed and approved by Patients signed an informed consent approved by the Israeli Ministry of Health (Helsinki approval no. 3518/2004, NCT00287131). The patients/participants provided their written informed consent to participate in this study.

## Author Contributions

HW-M, GG, OI, and MB contributed conception and design of the study. HW-M, HB, ML, and OI performed experiments and acquired the data. HW-M and OI wrote the first draft of the manuscript. GG, JS, and MB revised it critically for important intellectual content. All authors contributed to the article and approved the submitted version.

## Funding

This study was supported by a research grant from the Israel Science Foundation (grant 1804/18).

## Conflict of Interest

The authors declare that the research was conducted in the absence of any commercial or financial relationships that could be construed as a potential conflict of interest.
