# Supplementary material for: Genetic Modification of Tumor-Infiltrating Lymphocytes via Retroviral Transduction
Source: Front Immunol. 2021 Jan 7;11:584148. doi: 10.3389/fimmu.2020.584148 (PMC7817656; doi:10.3389/fimmu.2020.584148)
Supplement: Supplementary file 1 [file Presentation_1.pptx]

## Slide 1
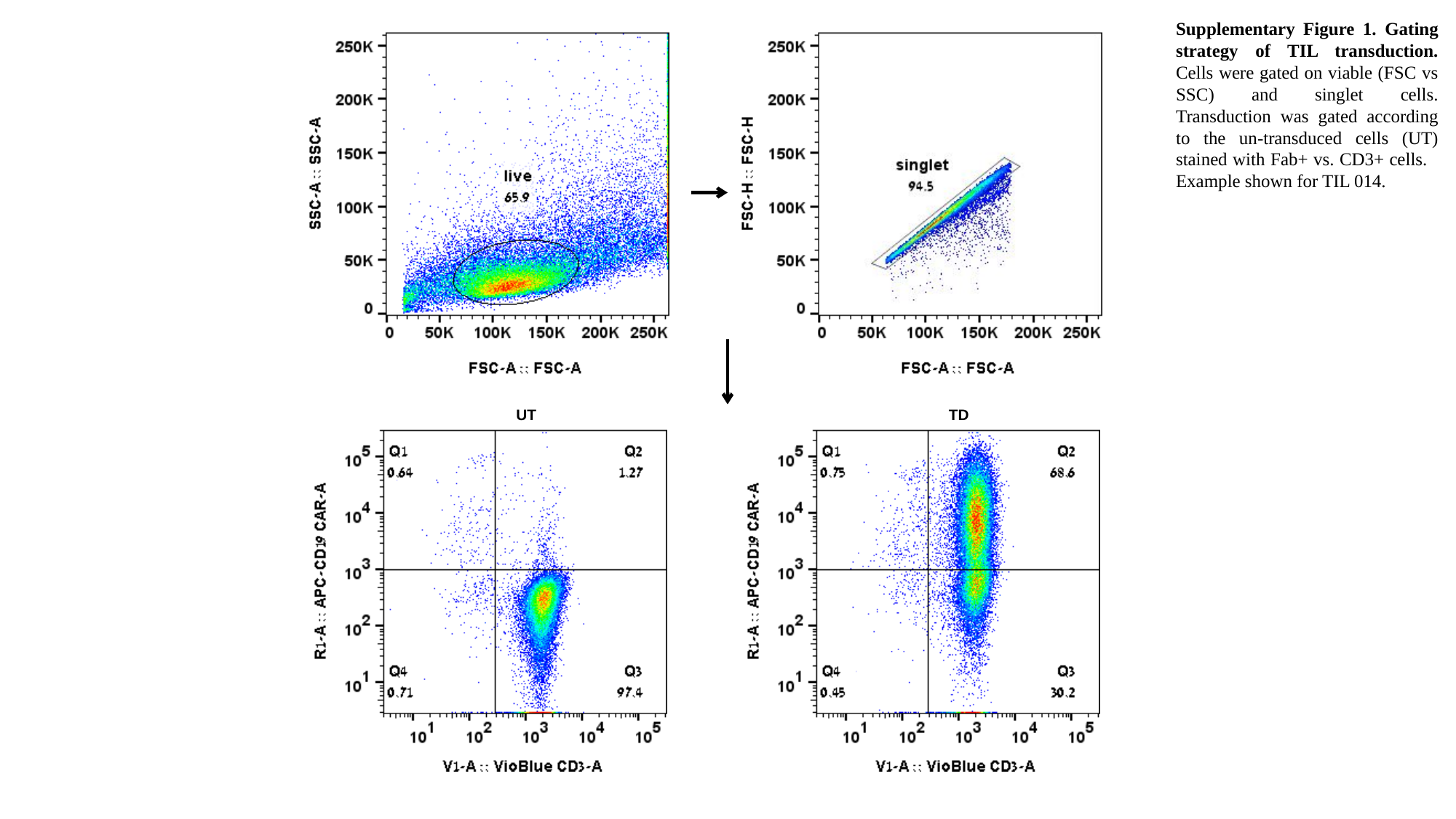

Supplementary Figure 1. Gating strategy of TIL transduction. Cells were gated on viable (FSC vs SSC) and singlet cells. Transduction was gated according to the un-transduced cells (UT) stained with Fab+ vs. CD3+ cells. Example shown for TIL 014.
TD
UT
