# Supplementary material for: Genetic Modification of Tumor-Infiltrating Lymphocytes via Retroviral Transduction
Source: Front Immunol. 2021 Jan 7;11:584148. doi: 10.3389/fimmu.2020.584148 (PMC7817656; doi:10.3389/fimmu.2020.584148)
Supplement: Supplementary file 2 [file Presentation_2.pptx]

## Slide 1
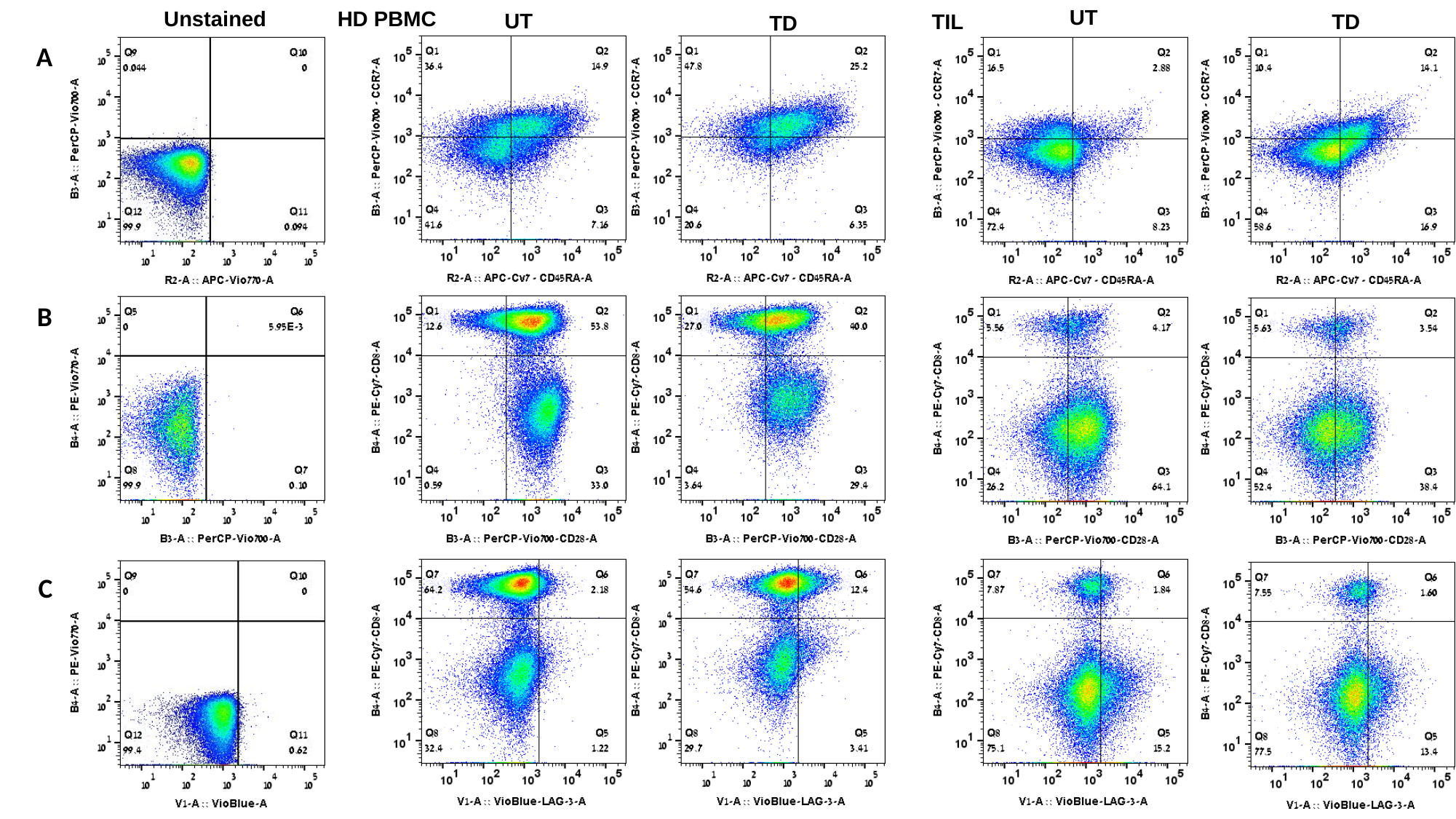

UT
HD PBMC
Unstained
UT
TIL
TD
TD
A
B
C

## Slide 2
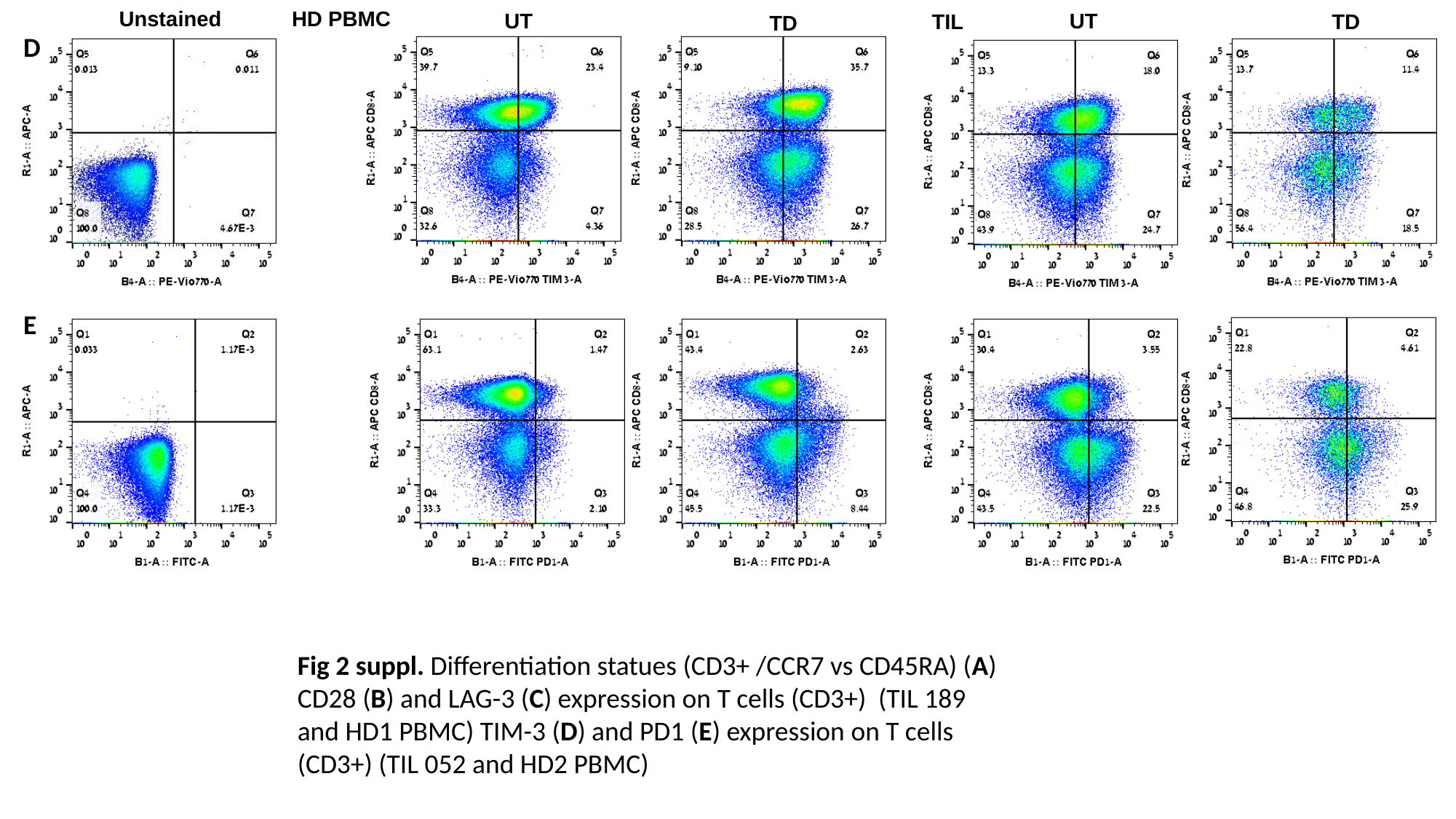

HD PBMC
Unstained
UT
UT
TIL
TD
TD
D
E
Fig 2 suppl. Differentiation statues (CD3+ /CCR7 vs CD45RA) (A) CD28 (B) and LAG-3 (C) expression on T cells (CD3+) (TIL 189 and HD1 PBMC) TIM-3 (D) and PD1 (E) expression on T cells (CD3+) (TIL 052 and HD2 PBMC)
